# Luteolin induces pyroptosis in HT-29 cells by activating the Caspase1/Gasdermin D signalling pathway

**DOI:** 10.3389/fphar.2022.952587

**Published:** 2022-08-29

**Authors:** Yiliu Chen, Shengsuo Ma, Dajin Pi, Yingchao Wu, Qian Zuo, Chongan Li, Mingzi Ouyang

**Affiliations:** School of Traditional Chinese Medicine, Jinan University, Guangzhou, China

**Keywords:** native compound, luteolin, pyroptosis, Gasdermin D, colon cancer

## Abstract

Luteolin, which is a natural flavonoid, has anti-inflammatory, antioxidant, and anticancer properties. Numerous studies have proven that luteolin inhibits the growth of many types of cancer cells by promoting apoptosis, autophagy, and cell cycle arrest in tumour cells. However, *in vivo* research on this topic has been limited. In addition, other studies have shown that luteolin exerts a good inhibitory effect on apoptosis-resistant cancer cells. While existing studies have not completely elucidated the mechanism underlying this phenomenon, we assume that luteolin, which is a natural compound that exerts its effects through various mechanisms, may have the potential to inhibit tumour growth. In our study, we proved that luteolin exerted a good inhibitory effect on the proliferation of colon cancer cells according to CCK8 and EdU fluorescence assays, and the same conclusion was drawn in animal experiments. In addition, we found that luteolin, which is an antioxidant, unexpectedly promoted oxidative stress as shown by measuring the levels of oxidative balance-related indicators, such as reactive oxygen species (ROS), SOD, H_2_O_2_ and GSH. However, the decreased oxidation of luteolin-treated HT-29 cells after treatment with the active oxygen scavenger NAC did not reverse the inhibition of cell growth. However, the Caspase1 inhibitor VX765 did reverse the inhibition of cell growth. Western blotting analysis showed that luteolin treatment increased the expression of Caspase1, Gasdermin D and IL-1β, which are members of the pyroptosis signalling pathway, in colon cancer cells. We further intuitively observed NLRP3/Gasdermin D colocalization in luteolin-treated HT-29 cells and mouse tumour tissues by immunofluorescence. These results suggest that luteolin inhibits the proliferation of colon cancer cells through a novel pathway called pyroptosis. This study provides a new direction for the development of natural products that inhibit tumour growth by inducing pyroptosis.

## Introduction

Colon cancer is one of the most common malignancies; it accounts for 10.2 and 9.2% of all cancer cases and cancer-related deaths worldwide, and these numbers represent the third and second highest numbers worldwide, respectively. With changes in diet, social pressures and an ageing population, the incidence of colon cancer is increasing year by year. According to the NCCN, China has the highest number of new CRC cases and CRC-related deaths in the world, and 387,600 new CRC cases and 187,100 CRC-related deaths were reported in 2015 alone. CRC has become a serious health problem (Benson et al., 2018) (Labianca et al., 2010). At present, surgery is the main strategy for treating early phase colon cancer. However, it is not easy to detect colon cancer in the early stage, so chemotherapy is a common approach for the comprehensive treatment of advanced colon cancer (Hohenberger et al., 2003; Labianca et al., 2010). However, many patients develop resistance to chemotherapy drugs, leading to treatment failure (Kozovska et al., 2014; Zhang C. C et al., 2019). Thus, it is critical to explore new strategies to better understand tumour biology and thereby improve clinical survival.

Pyroptosis, which is a form of programmed cell death (PCD), was discovered in recent years and is characterized by rapid damage to the plasma membrane. Recently, an increasing number of studies have shown that pyroptosis plays an important role in inhibiting the proliferation and growth of tumours *in vitro* and *in vivo* (Xia et al., 2019; Fang et al., 2020; Yu et al., 2021). Compared with other forms of cell death, pyroptosis has a distinct morphology and mechanism and is caused by two pathways: the canonical inflammasome pathway and the noncanonical inflammasome pathway. In the canonical inflammasome pathway, the inflammasome and pro-caspase 1 bind to the inflammatory complex *via* ASC and then activate caspase 1. Caspase 1 participates in the cleavage of IL-1β, Il-6 and Gasdermin D (GSDMD). The N-terminal fragment of GSDMD (GSDMD-NT) then translocates to the membrane, causing cell swelling and pore formation, leading to cytoplasmic outflow and resulting in cell membrane rupture and cell pyrosis. In the noncanonical inflammasome pathway, Caspase-4 and Caspase-11 are directly stimulated by intracellular LPS from Gram-negative bacteria, which activates and hydrolyses their protease activity. Activated Caspase-4 and Caspase-11 can act on GSDMD and produce the same cleavage products as Caspase-1; thus, signalling cascades promote pyroptosis *via* the release of LDH through the membrane channels formed by GSDMD-NT. Moreover, recent studies have shown that certain stimulations cause the activation of caspase-3 and then the cleavage of gasdermin E (GSDME), resulting in pyroptosis (Kovacs and Miao, 2017; Shi et al., 2017).

At present, NLRP3 inflammasome assembly, Caspase1 activation, and IL-1β secretion are the most characteristic and well-studied pathways associated with pyroptosis. Several investigations have shown that specific ions, compounds, or chemotherapeutic medications, such as iron, docosahexaenoic acid (DHA), cisplatin, paclitaxel, and doxorubicin, can cause GSDMD/GSDME-mediated pyroptosis in a variety of cancer cells (Zhou et al., 2018; Zhang H. L et al., 2019; Zeng et al., 2019; Zheng et al., 2020; Zhang et al., 2021). However, various challenges must be overcome during the administration of small molecules, such as rapid clearance from blood circulation, nonspecific biodistribution, and systemic adverse effects produced by high drug dosage. Natural products are a valuable source for the development of medications because of their vast availability and low toxicity (Gairola et al., 2021). Many natural medicines from traditional Chinese medicine (TCM) have been proven to have effective antitumor effects and a high level of safety and thus have become a major focus of antitumor medication research and development. Studies on the mechanism by which natural products exert antitumor effects currently focus on causing tumour cell apoptosis, arresting the cell cycle, increasing autophagy, and other similar mechanisms (Ma et al., 2021). The natural substance polyphyllin VI has recently been discovered to inhibit tumour growth by causing pyroptosis in non-small cell lung cancer cells (Teng et al., 2020). This research also provides a new understanding of the mechanisms by which natural compounds exert their antitumor effects.

One of the most prevalent natural products is luteolin (3,4,5,7-tetrahydroxyflavonoid), which is a flavonoid found in a number of plants, including Chinese herbs, such as honeysuckle and chrysanthemum, and vegetables, such as beets and cabbage. In breast cancer cells, liver cancer cells, drug-resistant gastric cancer cells, pancreatic cancer cells, and leukaemia cells, luteolin prevents tumour growth by reducing angiogenesis, inducing apoptosis, and arresting the cell cycle. The majority of research on luteolin’s antitumor properties has been performed *in vitro*, and only a few *in vivo* investigations have been conducted (Lin et al., 2008; Imran et al., 2019). Luteolin inhibits tumour cell proliferation, induces cell cycle arrest, increases intracellular reactive oxygen species (ROS) formation, and induces apoptosis in colon cancer cells *via* many signalling pathways (Chen et al., 2018; Yoo et al., 2022). The protein GSDMD is crucial to pyroptosis, and it can be activated by luteolin, according to this study. We also discovered that the luteolin-mediated activation of GSDMD, which is a crucial pyroptosis protein, was linked to the NLRP3/CASPASE1/GSDMD signalling axis. Thus, this study illustrated a new mechanism by which luteolin inhibits colon cancer for the first time, and these results will be used to develop this promising natural compound into new treatments for colon cancer.

## Materials and methods

### Protocol for cell culture and treatment

HT-29 cells were kindly provided by lecturer Jing Wang (School of Traditional Chinese Medicine, Jinan University, Guangzhou, China) and were grown in Dulbecco’s modified Eagle medium (DMEM) (Gibco Laboratories, United States) supplemented with 10% foetal bovine serum (FBS) and 1% penicillin and streptomycin at 37°C in a 5% CO_2_ atmosphere. The medium was replenished every 3 days, and when the cell density reached 80–90%, the cells were digested with 0.25% trypsin. For the subsequent investigations, HT-29 cells were seeded in six-well plates or 96-well plates and treated as described.

In 6-well plates, 5 × 10^5^ cells were seeded per well, whereas in 96-well plates, 5 × 10^3^ cells were seeded per well. Before treatment, the cells in the experimental group were grown overnight in minimum essential medium supplemented with 2% FBS until the cell density on the plates reached 50–60%. The cells were then treated with 50 μM, 100 μM, and 150 μM luteolin for 24 h, followed by 10 μM VX765/5 mM NAC for 2 h in serum-free media. VX765, which is a Caspase1 inhibitor from Sigma (United States), was dissolved in DMSO and utilized at a concentration of 10 μM, while the ROS inhibitor N-acetylcysteine (NAC, Sigma, United States) was used at a concentration of 10 mM in sterile deionized water.

### Tumour transplantation in nude mice

Thirty-six four-week-old BALB/C nude mice were obtained and housed in Jinan University’s SPF animal house, and these experiments were approved by the University’s Animal Ethics Committee (No. 2021830-02). By random number method, 10 mice were allocated to tumour control group, luteolin treatment group, and luteolin and VX765 combined treatment group, and the other 6 mice were intraperitoneal injection with 50 mg/kg/day of luteolin without tumour inoculation for subsequent toxicity test. PBS was used to suspend HT-29 cells that were in good growth condition. A total of 2 × 10^6^ HT-29 cells/100 µl were subcutaneously implanted into the right axilla of each nude mouse. The tumour volume was assessed after 3 days, and the weight of the nude mice was recorded. When the mean tumour volume reached 80 mm^3^, treatment began. The VX765 group was intraperitoneally injected with 50 mg/kg/day luteolin and 10 mg/kg/day VX765, and the control group was intraperitoneally injected with 200 µl normal saline and 50 mg/kg/day luteolin. Every 2 days, the nude mice were weighed, and the volumes of the transplanted tumours were measured. Fourteen days after injection, all the mice were euthanized, and the transplanted tumours were excised, weighed, and photographed. A portion of the tumour tissue was stored in liquid nitrogen for Western blotting analysis and oxidative stress-related marker expression assessment, while another portion was stored in 4% paraformaldehyde for staining.

### Cell viability evaluation

CCK-8 kits (K1018, APExBIO, United States) were used to assess the viability of HT-29 cells. Cells at approximately 90% confluence in 96-well plates were treated with various doses of luteolin for 24 h. A total of 10 μL of CCK-8 reagent was added to each well and incubated for 1 h at 37°C in the dark. A PerkinElmer microplate reader was used to measure the absorbance at 450 nm (PerkinElmer VIC-TOR 1420, United States).

### Clone formation experiment

A total of 1000 HT-29 cells were seeded in each well of 6-well plates. The cells were treated with different doses of luteolin (0 μM, 50 μM, 100 μM, or 150 μM) after adhesion. The medium was withdrawn and replaced with complete medium 24 h later. During this time, the culture medium was refreshed every 3 days. The cultured cells were collected 2 weeks later, washed once in PBS, and fixed for 20 min at room temperature with 4% paraformaldehyde. Then, each well was washed 3 times with 1 ml PBS. After staining with 1% crystal violet (C0121, Beyotime Biotechnology, China) for 30 min at room temperature, the cells were washed three times with PBS before being viewed and photographed under an inverted microscope. Image-Pro Plus was used to count the number of clones in each group, and every group with a number of cells greater than 15 was considered a clone.

### ELISA assay

ELISA (MM-0039M1, MEIMIAN, China; ABS510002-96T, Absin, China) was used to determine the concentrations of cytokines. The levels of interleukin-1 in cells and animal serum were measured using the method described in the manufacturer’s instructions. The EPOCH-2 Micropalte Reader was used to measure the absorbance of the obtained cell supernatant.

### Flow cytometry analysis

The proportion of apoptotic cells, the concentration of intracellular ROS, and changes in mitochondrial membrane potential were all measured by flow cytometry. The cells were seeded in 6-well plates, where they adhered to the well and were incubated for 24 h in media with various doses of luteolin (0 μM, 50 μM, 100 μM, or 150 µM), luteolin + NAC (5 mM), and luteolin + VX765 (10 mM). To generate a single cell suspension, the cells were digested with trypsin and centrifuged. According to the kit’s instructions, the fraction of apoptotic cells was determined (556570, BD Pharmingen, United States). The change in FITC fluorescence intensity was quantified according to the kit instructions to estimate the change in the intracellular ROS levels (CA1410, Solarbio, China). The mitochondrial membrane potential was measured following the instructions of the kit (M8650, Solarbio, China).

### Colorimetric assay for measurement of intracellular H_2_O_2_, SOD, and GSH levels

HT-29 cells were seeded in 6-well plates and treated with different stimuli. Then, the collected cells were disrupted with ultrasound and centrifuged at 14,000 rpm for 15 min at 4°C. The supernatant was used for the colorimetric determination of SOD activity (S0101S, Beyotime Biotechnology, China) and H_2_O_2_ (BC3595, Solarbio, China) and GSH (S0053, Beyotime Biotechnology, China) contents. The experimental operation was carried out according to the manufacturer’s instructions.

### Immunofluorescence staining

Immunofluorescence was used to examine the expression and interaction of NLRP3 and GSDMD in cells and animal tumour tissues. In summary, after fixation with 4% paraformaldehyde, the samples were washed three times with PBS for a total of 15 min and then blocked with 10% goat serum for 1 hour. An anti-NLRP3 antibody (1:600) was added and incubated for 6 h, the samples were washed three times with PBS, and then, the samples were incubated for another 10 h with an anti-GasderminD antibody. Then, the samples were washed with PBS three times and incubated for 50 min at room temperature. Finally, 50 µl DAPA + 150 µl PBS was applied to clean the samples three times before being photographed and observed under a fluorescence microscope.

### Western blotting analysis

After being subjected to different treatments, the cells were collected and lysed on ice for 20 min with RIPA buffer containing a mixture of 1% protease and 1% phosphatase inhibitor (Beyotime, China). Ultrasonic homogenization was performed with 5% power for 5 s, followed by stopping for 10 s, and the cycle was repeated 4 times. The samples were placed on ice for 5 min and centrifuged at 4°C at 12000 RPM for 6 min to collect the supernatants. The protein concentrations were quantified by a BCA protein assay kit (Beyotime Biotechnology, China). Protein (30 μg) was added to each well of a 10% polyacrylamide gel, separated by SDS‒PAGE and transferred to a polyvinylidene fluoride (PVDF) membrane. At room temperature, the membranes were blocked with Quick Block™ Blocking Buffer for 1 h and incubated with primary antibodies against NLRP3 (1:1000), ASC (1:1000), Caspase1 (1:1000), cleaved Caspase1 (1:1000), IL-1β (1:1000), GSDMD (1:1000), cleaved GSDMD (1:1000), β-actin (1:1000), and GAPDH (1:1000) at 4°C overnight (CST, United States). The membranes were then washed and incubated at room temperature with a fluorescent secondary antibody (1:5000, CST, United States) for 1 h. The membranes were washed with TBST 3 times for 5 min each. The strips on the membrane were visualized by a Bio-Rad ChemiDoc Touch imaging system, and the band intensities of the proteins of interest were quantified by ImageLab software.

### Statistical analysis

All the statistical analyses were performed by using SPSS 19.0 statistical software (IBM, United States), and the data are presented as the mean ± SD. One-way ANOVA or two-way ANOVA followed by Tukey’s post-hoc test was used to analyse the differences among experimental groups. *p* values < 0.05 were considered to be statistically significant.

## Results

### Luteolin inhibits the proliferation of HT-29 cells

**FIGURE 1:**
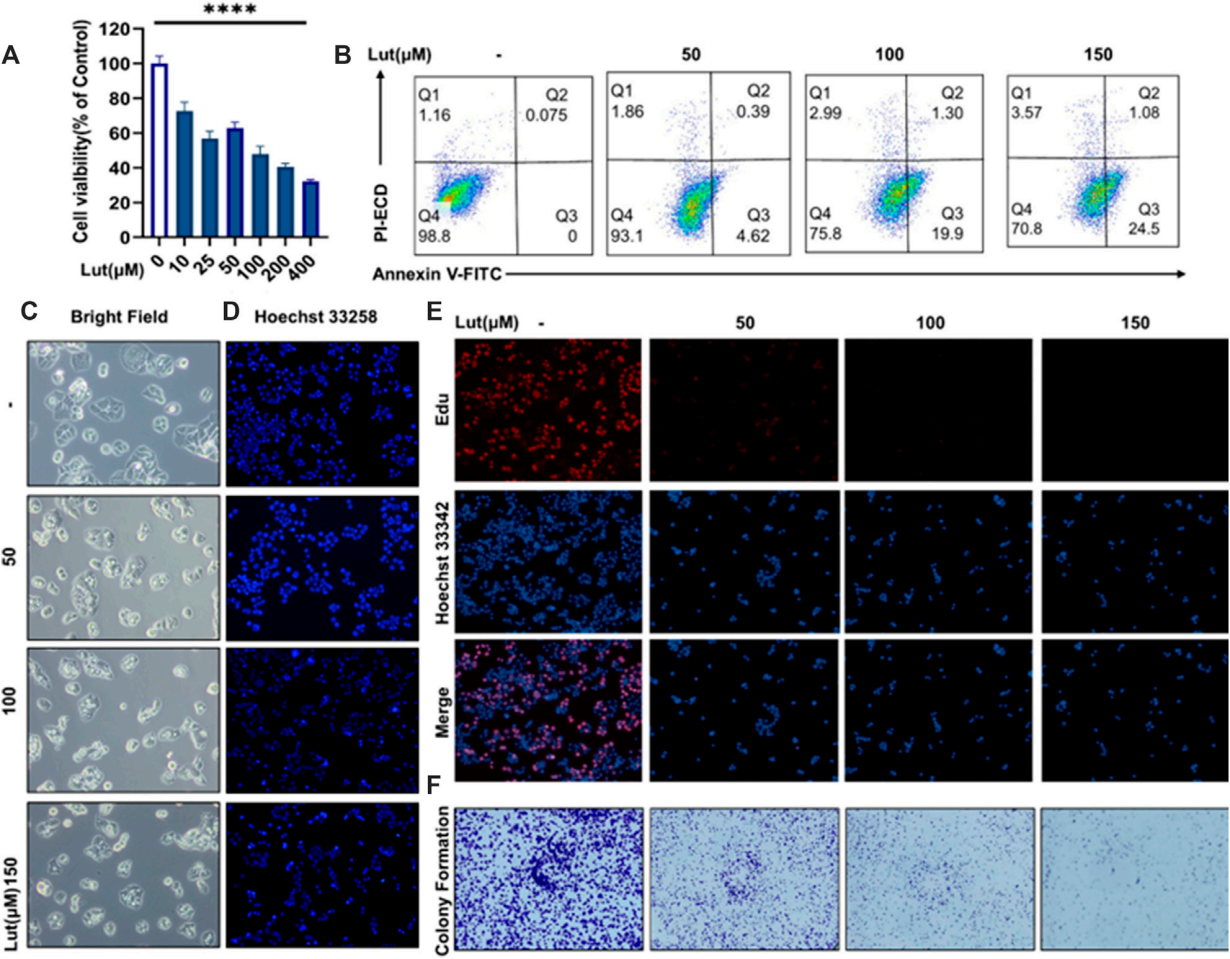
The effect of luteolin on the viability of HT-29 cells after 24 h of treatment. **(A)** Cells were treated with different concentrations of luteolin (10 μM, 25 μM, 50 μM, 100 μM, 200 μM and 400 μM), and the effect of luteolin on HT-29 cell inhibition was measured by CCK8. The results are presented as the mean ± SEM, *n* = 6. *p < 0.05, **p < 0.01, and ****p < 0.0001 vs. the control group. **(B)** Apoptosis was analysed by Annexin V‐FITC/PI staining and flow cytometry analysis. **(C)** Under a microscope, morphological alterations in the cells were observed. **(D)** Hoechst 33258 fluorescence staining was used to assess the changes in nuclear shrinkage of HT-29 cells treated with 0 μM, 50 μM, 100 μM and 150 μM luteolin for 24 h. **(E)** The effect of luteolin on the growth of colorectal cancer cells was studied using the EdU proliferation assay. Blue luminous cells indicate all of the cells, whereas red fluorescent cells are those in the S phase of mitosis. **(F)** Effects of different concentrations of luteolin on the colony formation of HT-29 cells.

To study the inhibitory effect of luteolin on the proliferation of colon cancer cells, we used the CCK8 method to determine the viability of different colon cancer cell lines, namely, the HT-29 (Figure 1A), SW620 (Supplementary Materials), HCT116 (Supplementary Materials) and RKO (Supplementary Materials) cell lines, after treatment with different doses of luteolin. Then, according to the experimental results of these CCK8 assays, we selected four concentrations of luteolin, 0 μM, 50 μM, 100 μM and 150 μM, and HT29 cells for subsequent experiments. The EdU labelling method was used to measure the proliferation of the cells (Figure 1E), in which red EdU staining indicates the proliferation activity of cells. Subsequently, we also used a method to measure the clone formation ability of cells (Figure 1F). The results of the CCK8, EdU staining and clone formation assays showed that luteolin had a strong inhibitory effect on the proliferation of HT-29 cells, and the inhibitory effect increased with increasing drug concentration. Annexin V-FITC-PI results showed increased cell numbers in the Q3 region, indicating the evagination of the cell membranes of luteolin-treated HT-29 cells, which was consistent with previous studies (Figure 1B). Hoechst 33258 staining showed nuclear atrophy after lignin treatment (Figure 1D). On the other hand, optical microscopy showed morphological changes in the membrane, such as vesiculation (Figure 1C), and the LDH contents in the cell supernatants were increased after luteolin treatment (Supplementary Figure S2), suggesting that luteolin may inhibit the tumour growth of HT-29 cells by triggering pyroptosis.

### Luteolin suppresses tumour growth by causing pyroptosis in HT-29 cells

**FIGURE 2:**
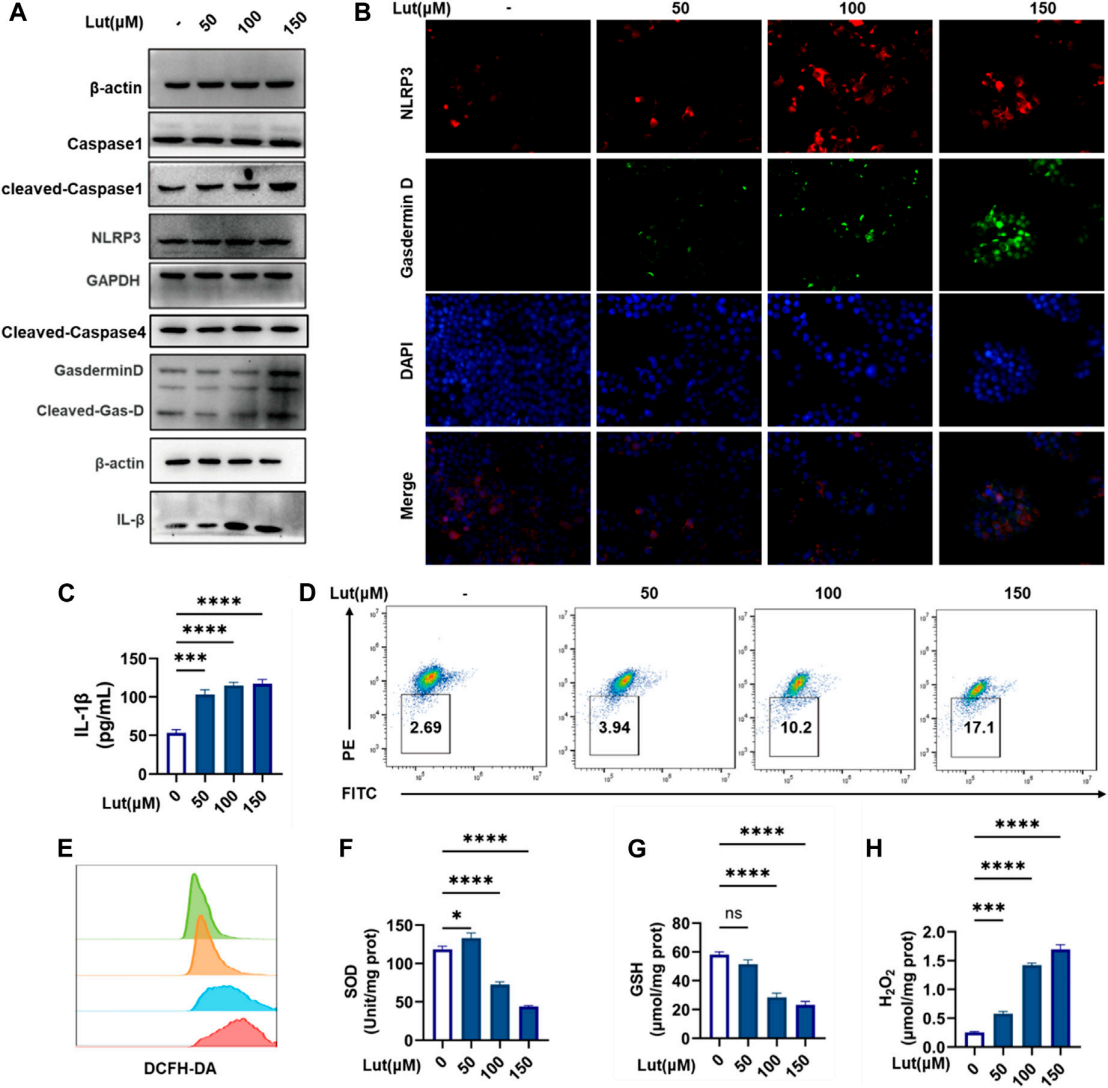
Luteolin induced pyroptosis and oxidative stress in HT-29 cells. **(A)** Control and luteolin-treated HT-29 cells were collected and analysed by WB to measure the expression levels of NLRP3, ASC, Pro-Caspase1, Caspase1, GSDMD, Cleaved-GSDMD (N-T), and IL-1β. GAPGH and β-actin served as internal controls. **(B)**. Immunofluorescence colocalization of NLRP3 with GSDMD in control and luteolin-treated cells. Cells were double labelled with antibodies specific for NLRP3 (Cy3) and GasderminD (FITC). DAPI was used to stain the nucleus blue. The cells that are labelled with three colours are those where pyroptosis occurred. **(C)** Control and luteolin-exposed HT-29 cells were harvested, and the IL-1β contents in cell supernatants were measured by ELISA. **(D)** The control and luteolin-treated HT-29 cells were harvested and analysed by flow cytometry to investigate the change in mitochondrial membrane potential. **(E)** Intracellular ROS production was assessed by flow cytometry. Green: control group, orange: 50 μM luteolin group, blue: 100 μM luteolin group, red: 150 μM luteolin group. **(F)** The SOD kit was used to measure the different SOD contents in cells. **(G)** The SOD kit was used to measure the different SOD contents in the cells. **(H)** The production of H_2_O_2_ in the cells of the control group and luteolin treatment group was measured. The results are presented as the mean ± SEM, *n* = 6. *p < 0.05, **p < 0.01, and ****p < 0.0001 vs. the control group.

Both the classical caspase-1-mediated pathway and the nonclassical caspase-4 (human)/caspase-11 (mouse)-mediated pathway can induce pyroptosis. Western blotting was used to measure the expression of cleaved-Caspase-1 and cleaved-Caspase-4, and the results showed that cleaved-capase-1 was activated, while the expression of cleaved Caspase-4 did not change significantly. We hypothesized that luteolin is dependent on the classical pathway of caspase-1 activation to induce cell pyroptosis. Then, Western blotting was used to assess key proteins in the classical pathway again, and the protein expression levels of NLRP3, GSDMD and IL-1β increased with increasing luteolin concentration (Figure 2A). When excessive ROS levels and other dangers threaten cell safety, the NLRP3 inflammasome forms and generates holes in the cell membrane by activating caspase-1, which cleaves and releases GSDMD, resulting in pyroptosis. Therefore, an immunofluorescence colocalizationapproach was used to assess the co-expression of the NLRP3 and GSDMD proteins in HT-29 cells. These two proteins were colocalized in luteolin-treated HT-29 cells (Figure 2B) and the increase of IL-β content in HT29 cell supernatant after luteolin treatment (Figure 2C), which further proved that luteolin induced the pyroptosis of HT29 cells through the classical pathway. ROS are involved in the activation of a series of signalling pathways that play key roles in the development and progression of cancer. Therefore, the levels of ROS, SOD, GSH and H2O2 were measured, and the results showed that luteolin could induce excessive ROS production and oxidative stress in HT29 cells (Figures 2E–H). Mitochondria are the main source of ROS, and the JC-1 marker showed decreased mitochondrial membrane potential, suggesting that excessive ROS production may occur due to mitochondrial damage in luteolin-treated HT-29 cells (Figure 2D).

### Effects of the reactive oxygen species scavenger NAC and Caspase1 inhibitor VX765 on cell viability after luteolin treatment

**FIGURE 3:**
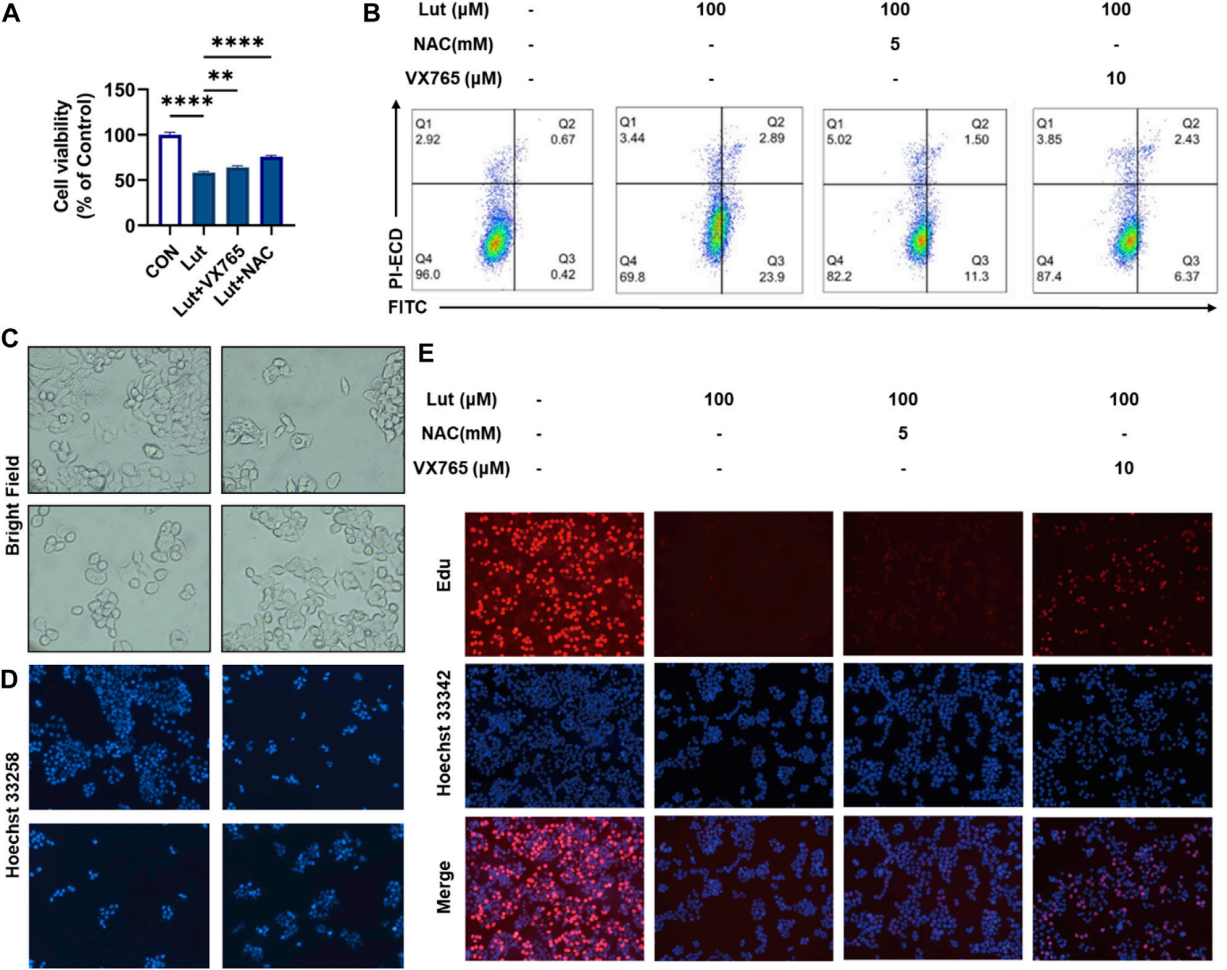
Effects of the ROS scavenger NAC and Caspase1 inhibitor VX765 on cell viability after luteolin treatment. **(A)** CCK8 assay was used to measure the viability of control HT-29 cells, luteolin-treated cells, NAC- and luteolin-treated cells, and VX765-and luteolin-treated cells. The results are presented as the mean ± SEM, *n* = 3. *p < 0.05, **p < 0.01, and ****p < 0.0001 vs. the control group. **(B)** Flow cytometry was used to analyse the changes in mitochondrial membrane potential among different treatment groups. **(C)** The morphological changes in the cells in different groups were observed under a microscope. **(D)** Hoechst 33258 fluorescence staining was used to observe the number of viable cells in different treatment groups. **(E)** The effect of luteolin, NAC and VX765 on the growth of colorectal cancer cells was studied using the EdU proliferation assay. Blue luminous cells indicate all the cells, whereas red fluorescent cells are those in the S phase of mitosis.

To investigate the roles of ROS and pyroptosis in inhibiting the proliferation of HT-29 cells, we treated cells with luteolin and with the ROS scavenger NAC and caspase 1-specific inhibitor VX765. CCK8, Annexin V-FITC/PI, Hoechst 33258 and EdU staining experiments showed that VX765 could reverse the inhibited growth of luteolin-treated HT-29 cells (Figures 3A,B,D,E). This reversal of luteolin-mediated HT-29 cell proliferation inhibition occurred mainly by inducing pyroptosis. Microscopy revealed that the morphology of cells treated with VX765 tended to be normal after luteolin treatment (Figure 3C).

### Both the antioxidant NAC and the pyroptosis inhibitor VX765 were able to alleviate the oxidative damage caused by luteolin in HT-29 cells

**FIGURE 4:**
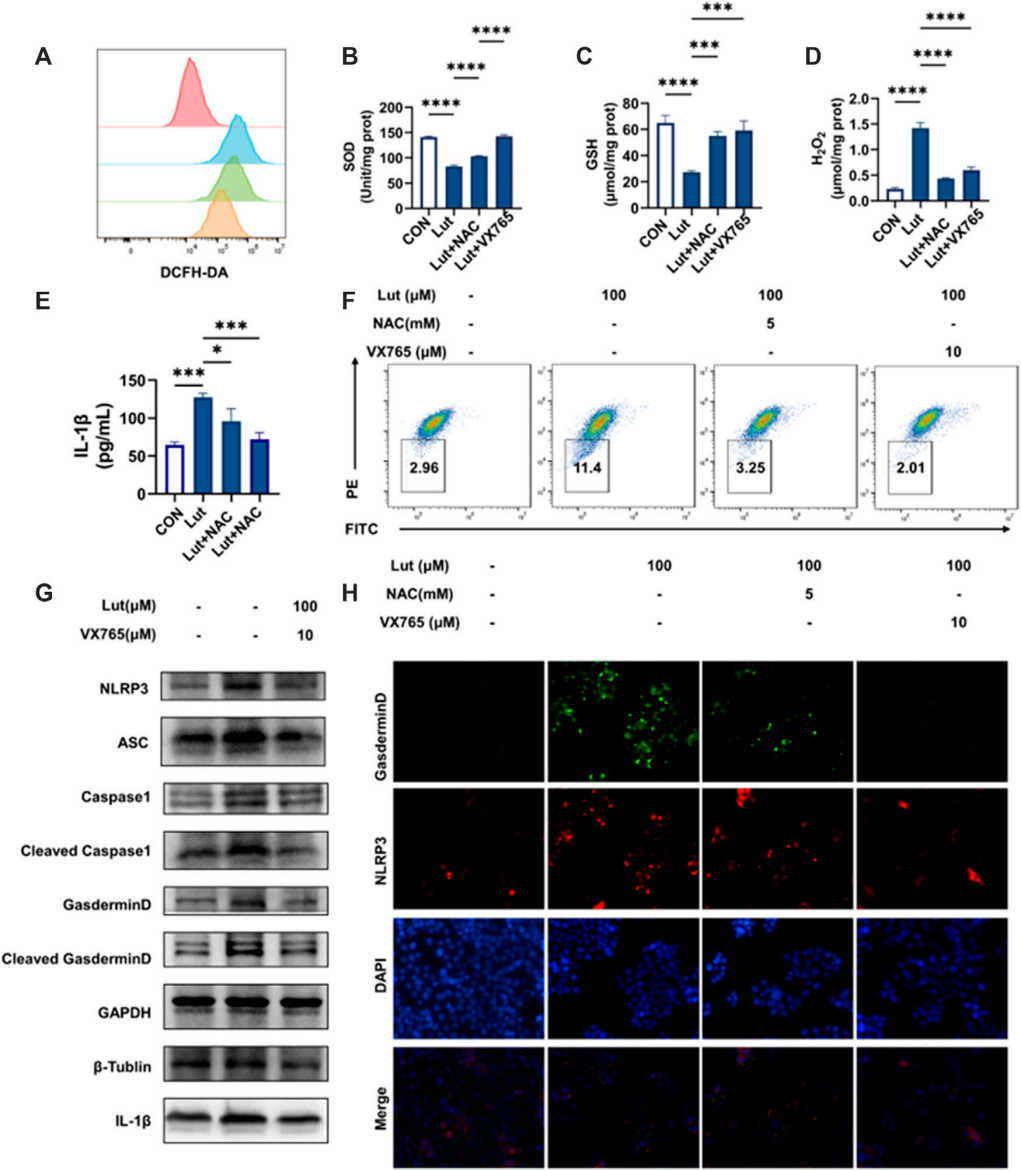
Effects of the reactive oxygen scavenger NAC and Caspase1 inhibitor VX765 on oxidative stress and pyroptosis after luteolin treatment. **(A)** Changes in intracellular ROS levels. Red: control group, Blue: luteolin group, Green: NAC group, Orange: VX765 group **(B)** Changes in SOD content. **(C)** Changes in GSH content. **(D)** Changes in H_2_O_2_ content. **(E)** The contents of IL-1β in the cell supernatants. **(F)** Changes in mitochondrial membrane potential. **(G)** The control, luteolin-treated and VX765-treated HT-29 cells were collected and analysed by WB to assess the expression levels of NLRP3, ASC, Pro-Caspase1, Caspase1, GSDMD, Cleaved-GSDMD (N-T), and IL-1β. GAPGH, β -Tubulin and β-actin served as internal controls. **(H)** Immunofluorescence colocalization of NLRP3 with GSDMD in the control and luteolin-treated cells. Cells were double stained for NLRP3 (Cy3) and GSDMD (FITC). DAPI was used to stain the nucleus. The cells that were labelled with three colours are those where pyroptosis occurred. The results are presented as the mean ± SEM, *n* = 3. *p < 0.05, **p < 0.01, and ****p < 0.0001 vs. the control group.

We further measured the levels of oxidative stress-related indicators in the cells cotreated with NAC or VX765 combined with luteolin and found that both agents could alleviate intracellular oxidative stress and reduce mitochondrial damage (Figures 4A–D,F), but the increase in IL-1β production and the expression of pyroptosis-related proteins could be inhibited only by VX765, not NAC (Figures 4E,G,H). These results suggested that luteolin caused pyroptosis and then further induced oxidative stress after mitochondrial damage.

### Luteolin inhibited tumour growth in xenograft mouse models by inducing pyroptosis

**FIGURE 5:**
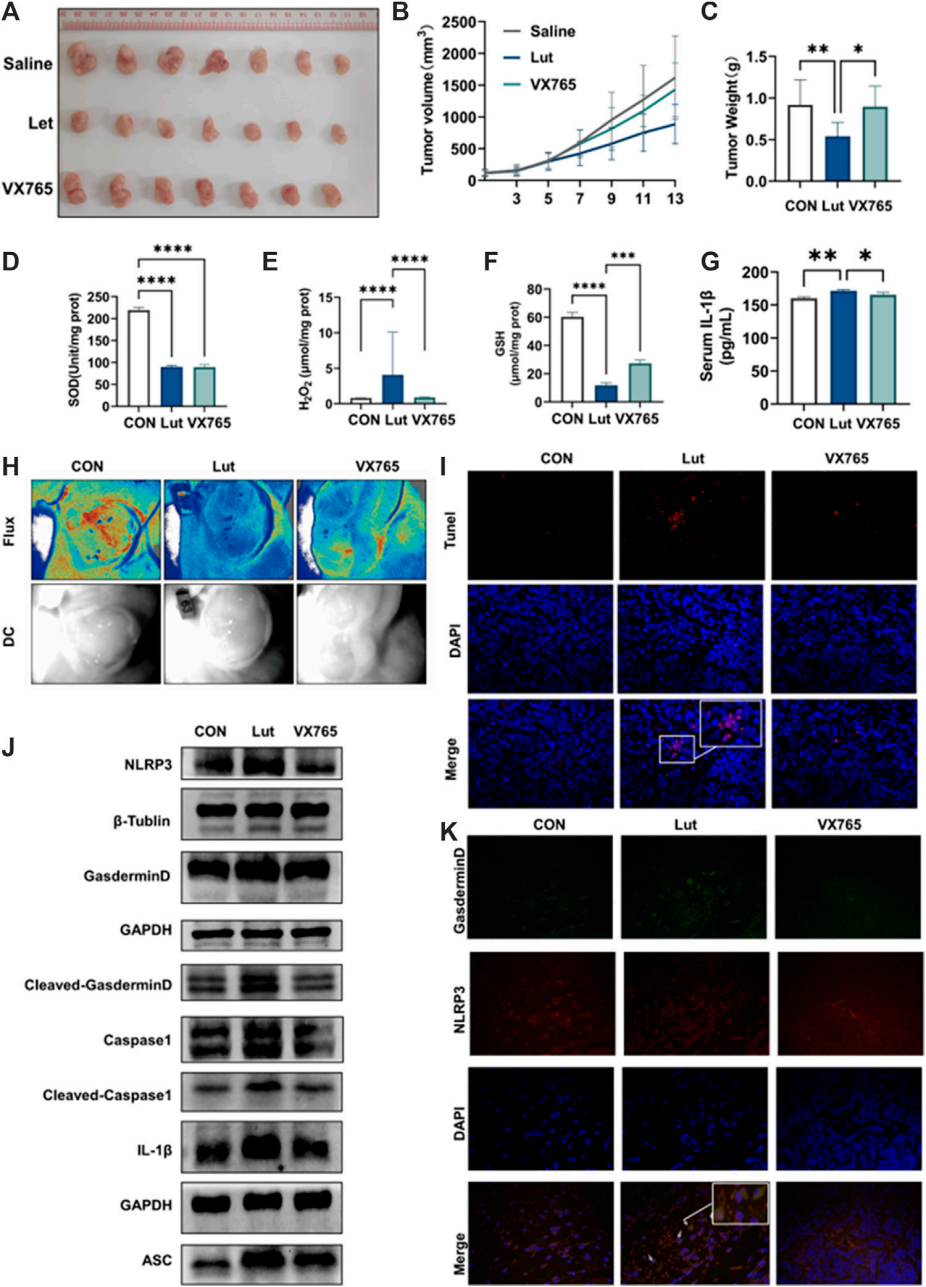
Effects of luteolin on tumours *in vivo*. **(A)** Tumours in mice. **(B)** Tumour volume was measured every 2 days. Tumour volume = 0.5× maximum diameter × minimum diameter squared. **(C)** Tumour weight. **(D)** SOD content in tumours. **(E)** H_2_O_2_ content in tumours. **(F)** GSH content in tumours. **(G)** The content of IL-1 in the serum of mice was determined by ELISA. **(H)** Image of angiogenesis in tumours. Red indicates blood flow. **(I)** TUNEL staining of tumour tissues **(J)** Tumour tissues of control, luteolin-, and VX765-treated mice were collected, and the expression levels of NLRP3, ASC, Pro-Caspase1, Caspase1, GSDMD, Cleaved-GSDMD (N-T), and IL-1β were analysed by WB. GAPGH and β-actin were used as internal controls. **(K)** Immunofluorescence colocalization of the NLRP3/GSDMD proteins in mouse tumour tissues.

We explored whether luteolin can inhibit tumours *in vivo* and the underlying mechanism. HT-29 cells were implanted into immunodeficient nude mice to evaluate the inhibitory effect of luteolin on colon cancer proliferation *in vivo* and the underlying mechanism. When the tumour had grown to 70–100 mm^3^, 0.1% DMSO or luteolin (50 mg/kg) was administered intraperitoneally once every other day for a total of seven times. Luteolin inhibited HT-29 tumour xenograft growth, which could be reversed by injecting VX765 (10 mg/kg) and luteolin together (every 2 days) (Figures 5A–C). Luteolin has been demonstrated to produce oxidative stress in cancers in animals (Figures 5D–G). Furthermore, luteolin prevented tumour angiogenesis (Figure 5H). *In vivo* investigations have demonstrated that luteolin therapy inhibits xenograft tumour development and is linked to pyroptosis pathway activation (Figure 5J). TUNEL staining of transplanted tumour tissues indicated that luteolin might cause apoptosis, while NLRP3/GSDMD colocalization staining indicated that luteolin could also cause pyroptosis (Figures 5I,K). These results suggest that luteolin can inhibit tumour growth through multiple pathways, among which pyroptosis plays a key role.

## Discussion

Colon cancer is a malignant tumour that originates from the colon mucosa epithelium and is caused by a variety of carcinogenic factors, such as environmental or genetic factors (Cappell, 2008; Labianca et al., 2010). It is one of the most common malignant tumours, and its incidence and mortality rates rank among the top five of cancers worldwide (Siegel et al., 2017). Furthermore, the population of people with colon cancer appears to be getting younger (Teng et al., 2019). Surgical treatment is currently the primary treatment for early colon cancer; however, because colon cancer is difficult to detect in the early stage, improved approaches for the treatment of colon cancer *via* combination therapy or chemotherapy have emerged (Cappell, 2008). There are many chemotherapeutic approaches used to treat colon cancer, including 5-fluorouracil (5-FU), calcium folate and capecitabine, orisplatin, and irinotecan, all of which have been shown to promote cell apoptosis and autophagy, thereby decreasing tumour development and cell proliferation (Pallis and Mouzas, 2006; Kajmolli et al., 2021). However, there have been few trials on promoting pyroptosis to stop colon cancer cells from proliferating. Two of the most common causes of poor treatment response include resistance to apoptosis and alterations in the tumour microenvironment (Huerta et al., 2006; Kozovska et al., 2014; Hu et al., 2016).

Pyroptosis is a type of PCD that is associated with inflammation and differs from apoptosis (Bertheloot et al., 2021). New research reveals that pyroptosis in tumour cells can prevent tumour cell proliferation (Wang et al., 2017). However, more research is needed to determine whether medications can cause tumour cells to undergo pyroptosis and whether drugs that cause pyroptosis play a role in the tumour process. Pyroptosis can be activated by GSDMA, GSDMB, GSDMC, GSDMD, and GSDME, which are all members of the Gasdermin superfamily, with GSDMD and GSDME being the most extensively studied in the context of cell pyroptosis (Kovacs and Miao, 2017). The Gasdermin superfamily of proteins has been found to have inherent necrotic activity. The Gasdermin-N domain is important; however, it is frequently covered by the Gasdermin-C domain. Gasdermin-N is released when sequences between the gasdermin-N and gasdermin-c domains are hydrolysed and cleaved. Oligomers are produced when the necrotic Gasdermin-N domain is transported to the plasma membrane. These oligomers create transmembrane pores on the membrane, causing cell membrane rupture, content leakage, cell enlargement, and many bubble-like protrusions. Pattern-recognition receptors (PRRs), such as Toll-like receptors (TLRs), Nod-like receptors (NLRs), and absent in melanoma (AIMs), recognize pathogen-associated molecular patterns (PAMPs) and damage-associated molecular patterns (DAMPs) to activate inflammasomes *via* the canonical inflammatory pathway (Vande Walle and Lamkanfi, 2016; Hsu et al., 2021; Yu et al., 2021). The NLRP3 inflammasome is the most well-studied of these inflammasomes. NLRP3 is generally maintained in an inactive but signalling component state within cells (Sharma and Kanneganti, 2021). When cells are injured or heterologous substances are detected, NLRP3 oligomerizes through its NACHT domain. Homotypic PYD-PYD interactions lead to the aggregation of PYD and the adaptor protein apoptosis-associated speck-like protein containing CARD (ASC). ASC has an N-terminal PYD domain and a C-terminal CARD domain, and the C-terminal CARD domain interacts with other CARD domains to assemble inflammatory cystease (Sutterwala et al., 2014; Seoane et al., 2020). As a result, inflammatory cypsin is firmly packed and activated to automatically generate active cypsin. In the case of ProCaspase1, the active cystease automatically cleaves to form a dimer of P10/P20 and eventually a 4-polymer, resulting in the activation of NLRP3’s executive protein Caspase1 (Sun et al., 2019). This cysteine-specific cleavage of Caspase1 not only causes cell death but also cleaves proinflammatory cytokines, including IL-1 and IL-18, as well as GSDMD (Qiu et al., 2019). Chrysophanol has been shown to promote MKN28 gastric cancer cell pyroptosis *via* NLRP3/Caspase1/GSDMD, and the Caspase1 inhibitor VX765 can reduce the chrysophanol-induced pyroptosis of MKN28 gastric cancer cells (Min et al., 2021). Furthermore, 3-(2-oxo-2-phenylethylidene)-2,3,6,7-tetrahydro-1H-pyrazino-[2,1-a], which is a novel small molecular activator of Nrf2, was proven to prevent the development of colorectal adenomas in AOM-DSS models by inhibiting NLRP3 inflammasome activation in the colon (Wang et al., 2016). As a result, the activation of Caspase1 inhibits cancer cell proliferation, and drugs that activate the NLRP3 inflammasome have the potential to be used as a treatment for colon cancer.

**FIGURE 6:**
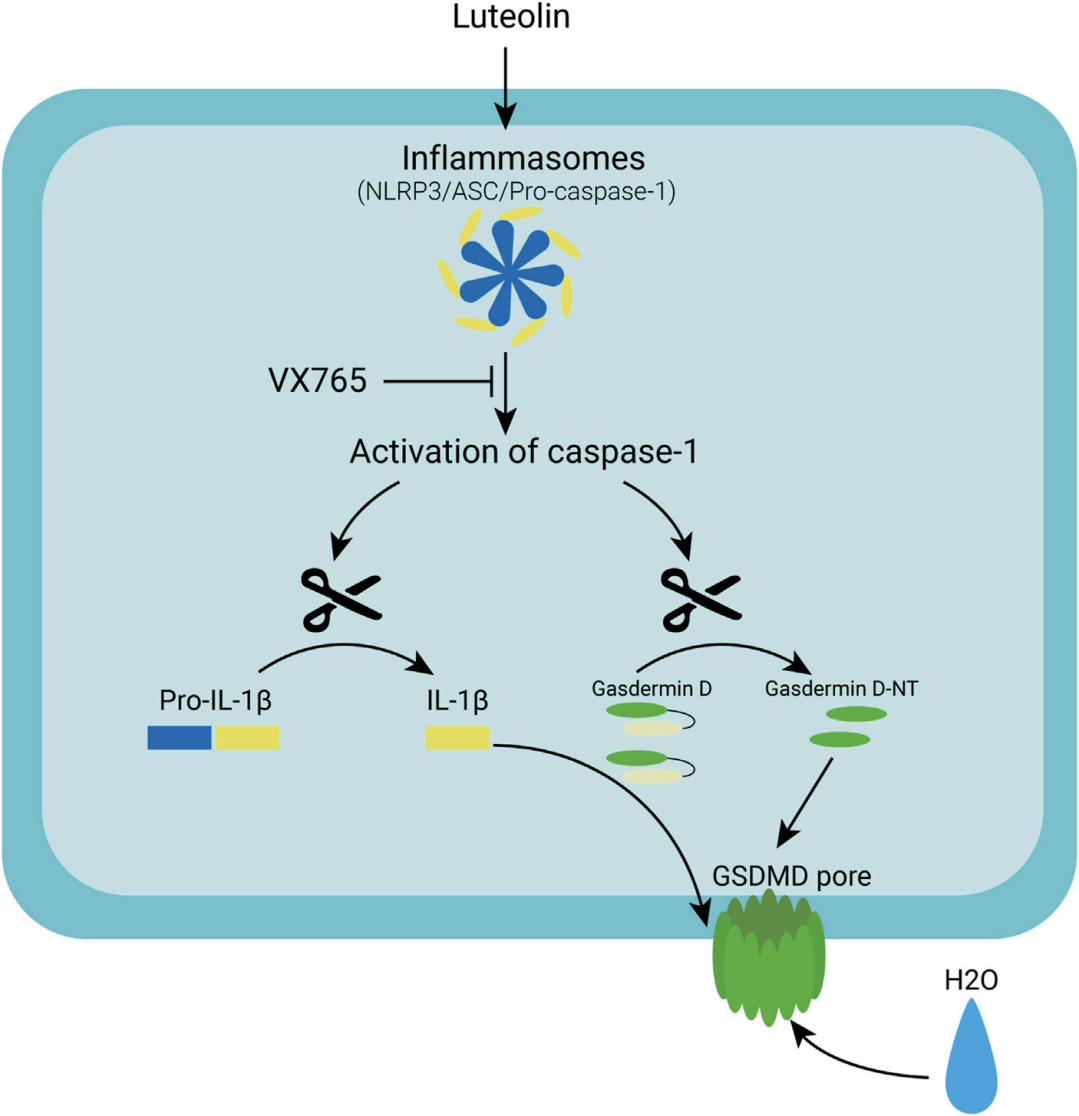
A schematic diagram of the molecular mechanism by which Luteolin induced pyroptosis *via* the activation of the NLRP3/Caspase-1/GSDMD signal axis in HT29 cells.

Many natural products from TCM have been found to have antitumor properties (Wang et al., 2021). Honeysuckle is a Chinese herbal medicine that has been shown to significantly reduce cancer cell proliferation and tumour growth. Luteolin isolated from honeysuckle has previously been shown to stop colon cancer from spreading by arresting the cell cycle in the G2/M phase (Chen et al., 2018). Luteolin has also been shown to inhibit colon cancer proliferation by inducing apoptosis (Yoo et al., 2022). According to other studies, luteolin inhibits the adaptation to hypoxia and progression of human colon cancer (Monti et al., 2020). The colon cancer cell line HT-29 was found to be inhibited by luteolin in this study. In patients treated with luteolin, both tumour volume and tumour size were significantly reduced x many small bubbles in the cell membranes of luteolin-treated HT-29 cells, which was consistent with the pyroptotic changes in cellular morphology. As a result, we hypothesized that luteolin inhibits tumour growth by causing pyroptosis in HT-29 cells. Using Western blotting analysis of pyroptosis-related proteins, we confirmed that luteolin increased the expression of pyroptosis-related proteins in colon cancer cells. Furthermore, we discovered that luteolin caused an increase in the hydrogen peroxide and superoxide levels. As a second biological signal, ROS are thought to be involved in NLRP3 activation. Natural products have been shown to activate NLRP3 by increasing ROS levels in tumour cells, which activates Caspase1, causing GSDMD to be cleaved and pyroptosis to occur. When inflammatory bodies are activated in macrophages, GSDMD causes mitochondrial depolarization and degradation, which leads to plasma membrane rupture, according to a recent study. GSDMD-N also translocates to the mitochondrial membrane and permeabilizes it, activating the Bcl-2-related X (Bax) apoptosis regulator, releasing cytochrome C (Cyt C), and triggering the Caspase-3-mediated mitochondrial apoptosis pathway, according to Rogers et al. (2019). However, we were unable to significantly reverse the reduction in cell proliferation caused by luteolin when we used the antioxidant NAC. We hypothesized that the increase in ROS caused by luteolin is not the main pathway of NLRP3 activation and that ROS production occurs due to the ’damage caused by the drug to tumour cell mitochondria. In the Jc-1 experiment, luteolin treatment caused mitochondrial damage in tumour cells (Figure 6). The mechanism by which luteolin causes colon cell death is unknown. We used molecular docking to investigate the mechanism by which luteolin inhibits growth and discovered that luteolin and NLRP3 strongly interact. We believe that luteolin causes pyroptosis in HT-29 colon cancer cells by directly activating NLRP3. Finally, luteolin causes HT-29 cell death by activating the NLRP3/Caspase1/GSDMD signalling axis, and this was shown here for the first time. These results support the development of luteolin as a novel therapeutic candidate for colon cancer.

## Data Availability

The raw data supporting the conclusion of this article will be made available by the authors, without undue reservation.
