# Supplementary material for: Luteolin induces pyroptosis in HT-29 cells by activating the Caspase1/Gasdermin D signalling pathway
Source: Front Pharmacol. 2022 Aug 29;13:952587. doi: 10.3389/fphar.2022.952587 (PMC9464948; doi:10.3389/fphar.2022.952587)
Supplement: Supplementary file 1 [file DataSheet1.PDF]

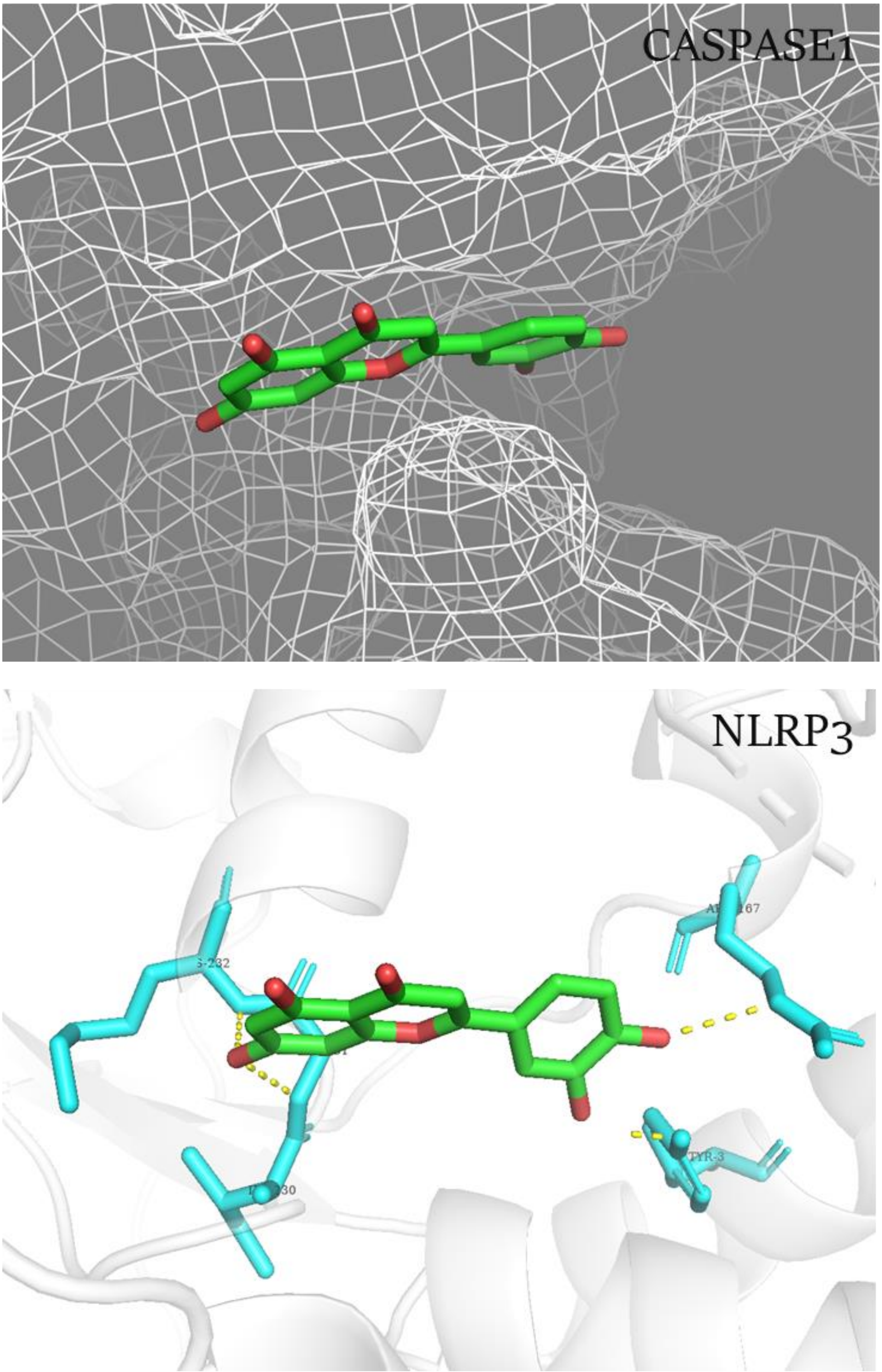
CASPASE1
NLRP3
TYR-3

Figures 1. Molecular docking results of Luteolin with different proteins. The optimal binding energy of Luteolin and Caspase-1 was -6.9KCal，The optimal binding energy of Luteolin and Caspase-1 was -8.1KCal.

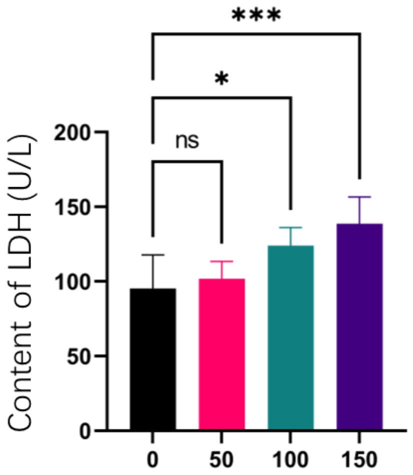

Figures 2. Changes of LDH content in supernatant of HT29 cells treated with luteolin at different concentrations (0 μM, 50 μM, 100 μM, 150 μM). The results are presented as the mean ± SEM, n = 6. *p < 0.05, **p < 0.01, and ****p < 0.0001 vs. the control group.

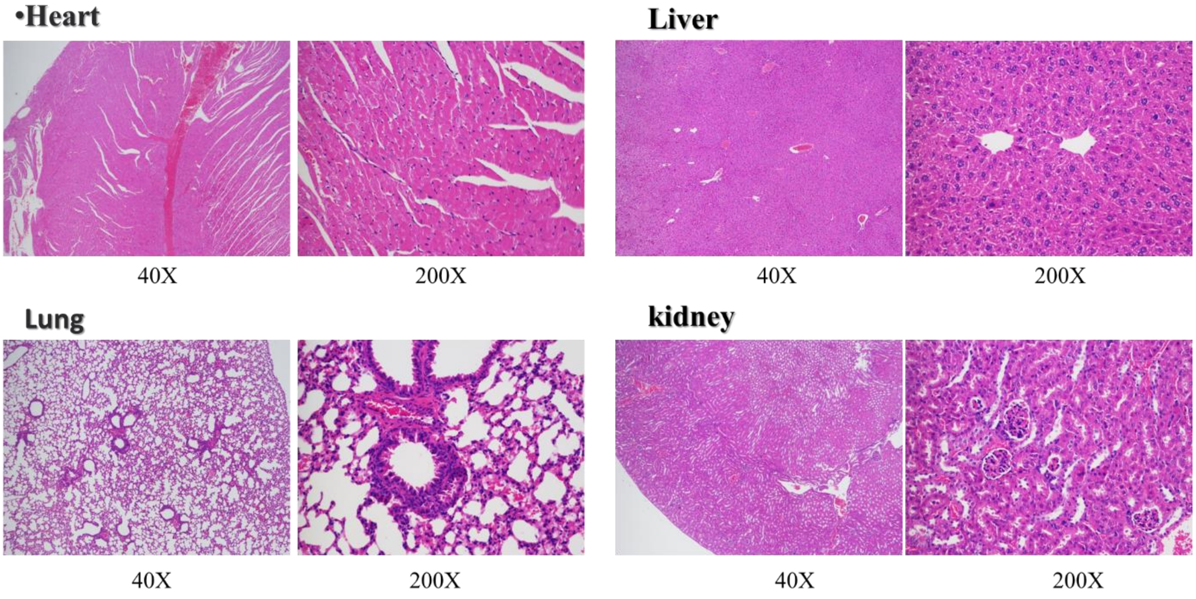

Figures 3. Pathological examination of different organs in mice after intraperitoneal injection of luteolin at 50mg/Kg/day

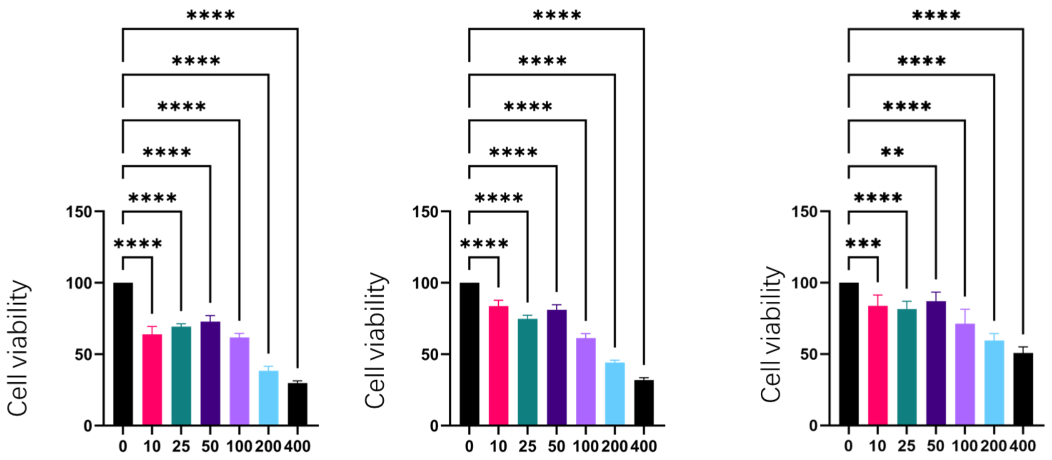

A B C

Figures 4. The effect of luteolin on the viability of colon cancer cells after 24 hours of treatment. Cells were treated with different concentrations of luteolin (10 μM, 25 μM, 50 μM, 100 μM, 200 μM and 400 μM), and the effect of luteolin on colon cancer cells inhibition was measured by CCK8. The results are presented as the mean ± SEM, n = 6. *p < 0.05, **p < 0.01, and ****p < 0.0001 vs. the control group. (A)HCT116 (B)SW620 (C)DLD1

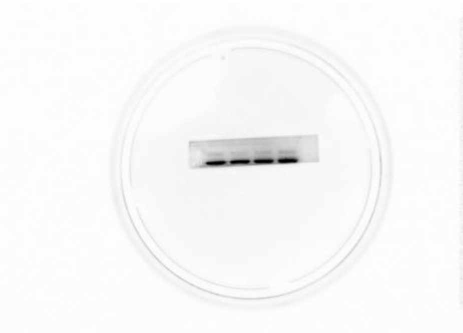

Caspase-1

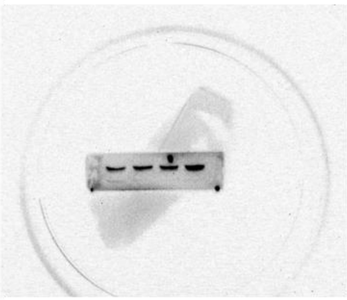

Cleaved-Caspase-1

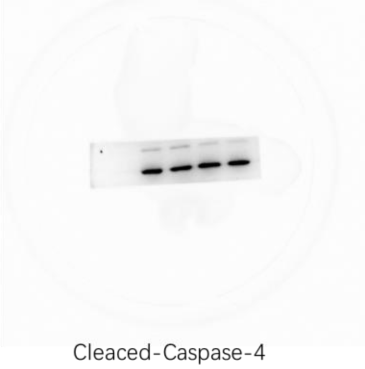

Cleaced-Caspase-4

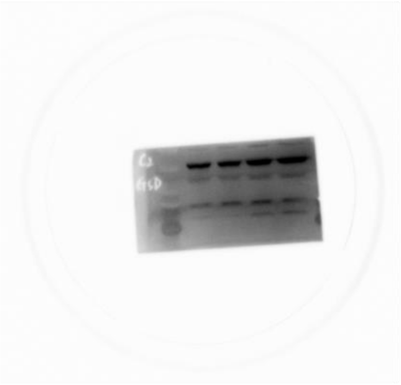

Gasdermin D

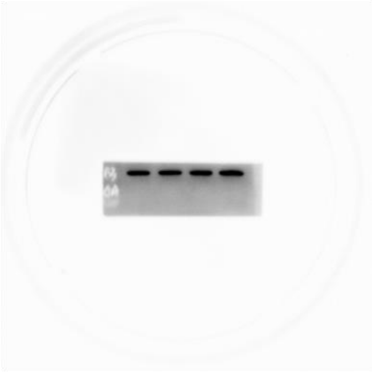

GAPDH

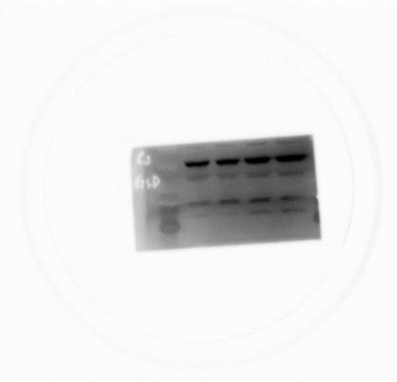

Cleaved-Gasdermin D

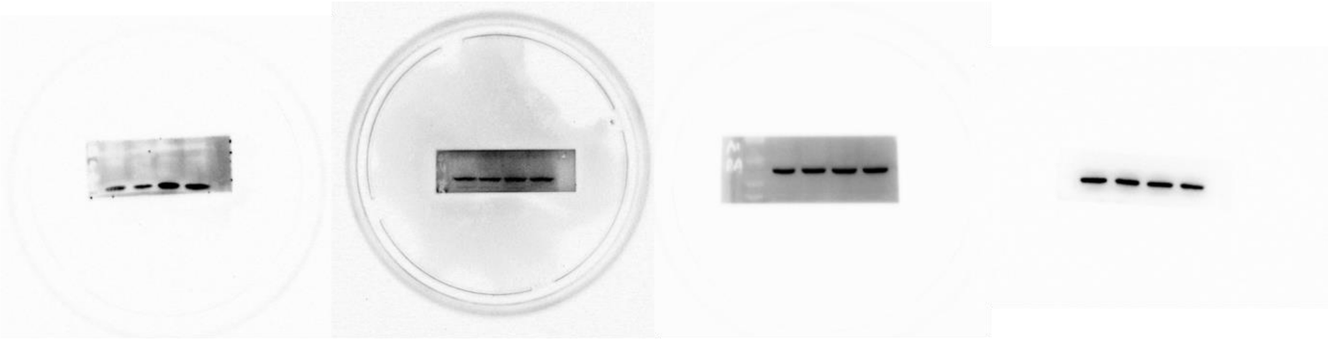

IL-1β NLRP3 β-actin-1 β-actin-2

Figures 5. Uncropped western blot of the original image of Figures 2A in the paper.

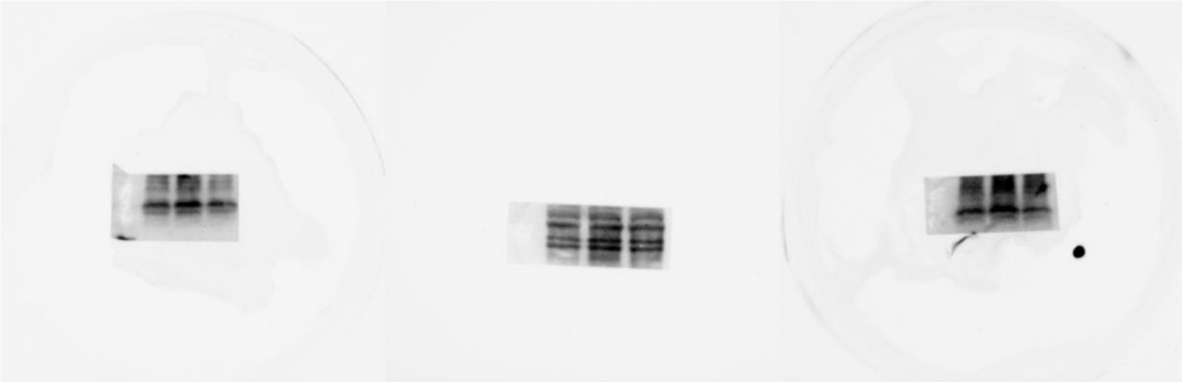

ASC Caspase-1 Cleaved-Caspase-1

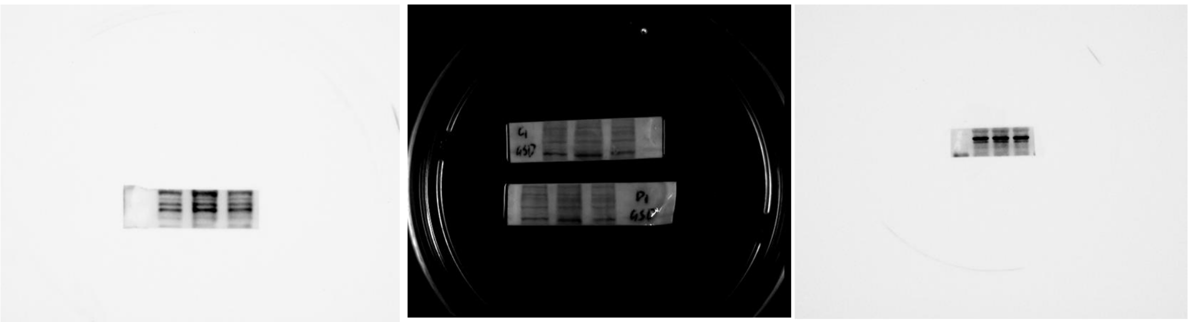

Cleaved-Gasdermin D Gasdermin D GAPDH

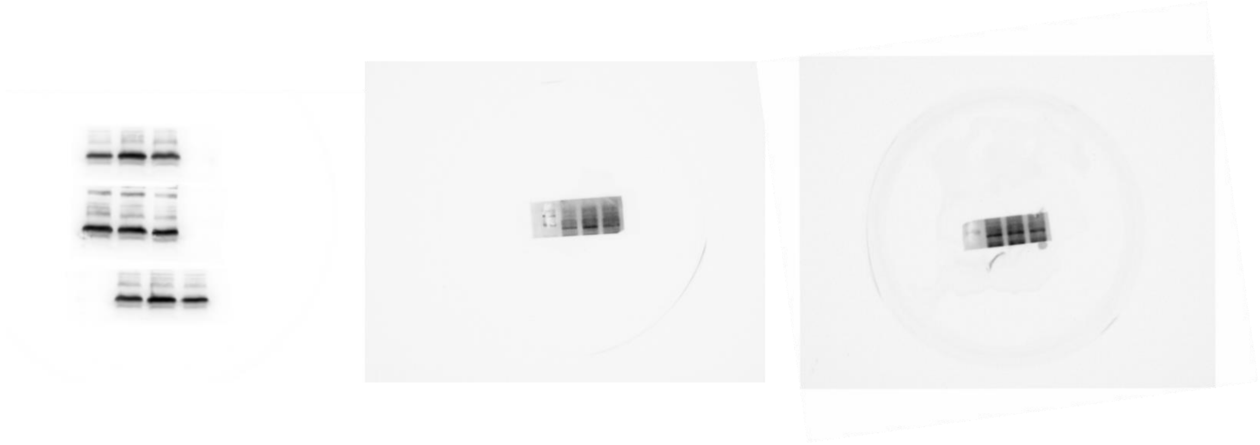

IL-1β NLRP3 β-tublin

Figures 6. Uncropped western blot of the original image of Figures 4A in the paper.

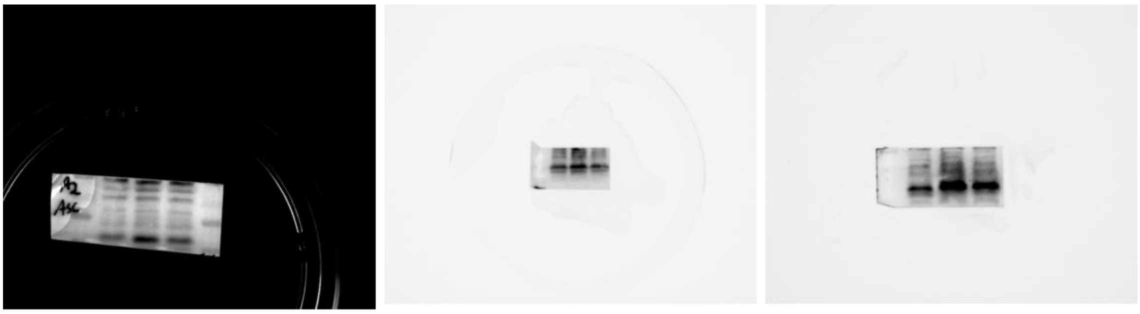

ASC-1 ASC-2 ASC-3

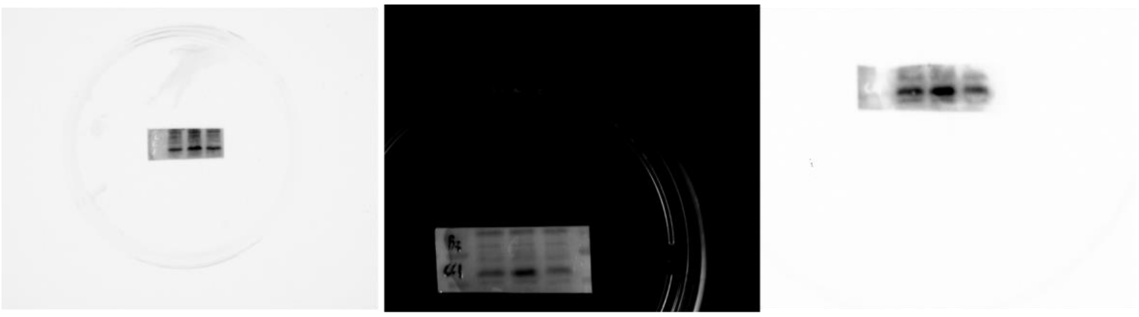

Cleaved-caspase-1 Cleaved-caspase-1 Cleaved-caspase-1

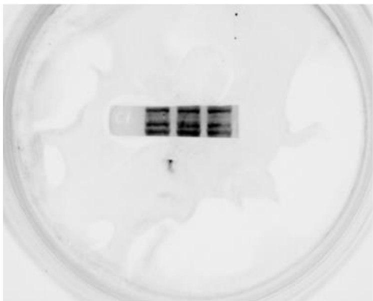

Caspase-1

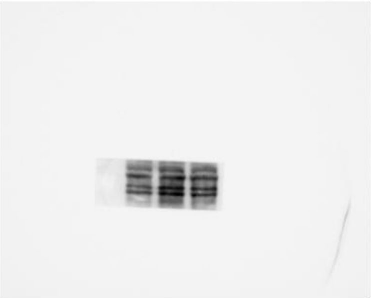

Caspase-1

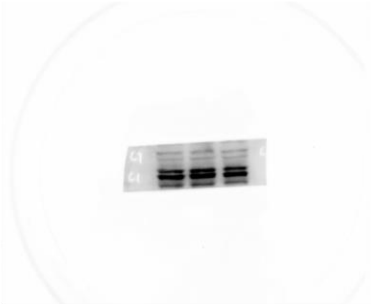

Caspase-1

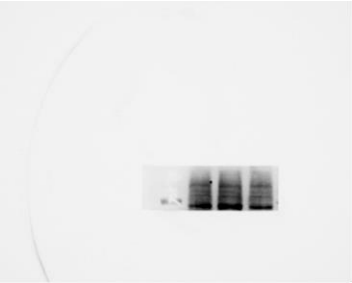

NLRP3

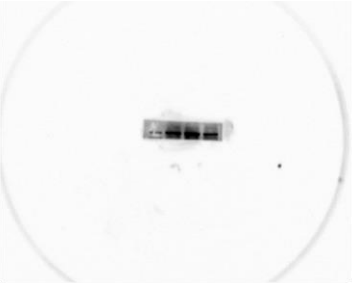

NLRP3

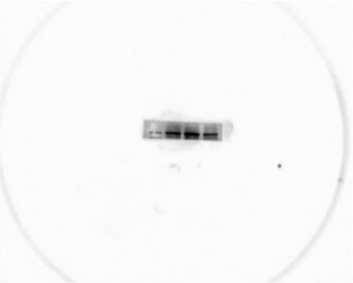

NLRP3

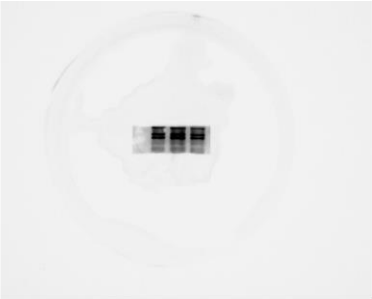

Cleaved GasderminD

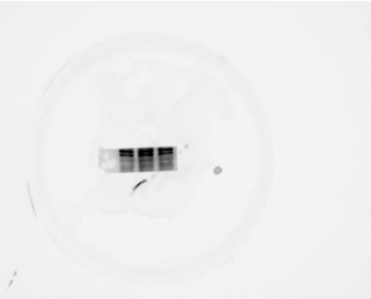

Cleaved GasderminD

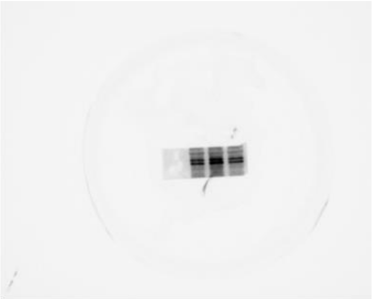

Cleaved GasderminD

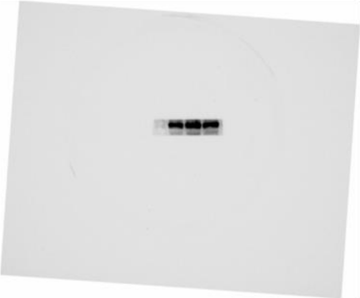

Gasdermin D

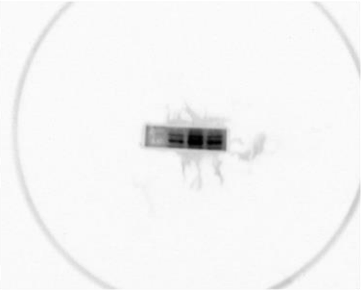

Gasdermin D

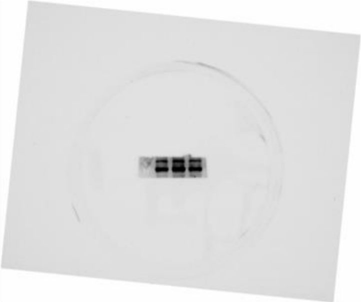

Gasdermin D

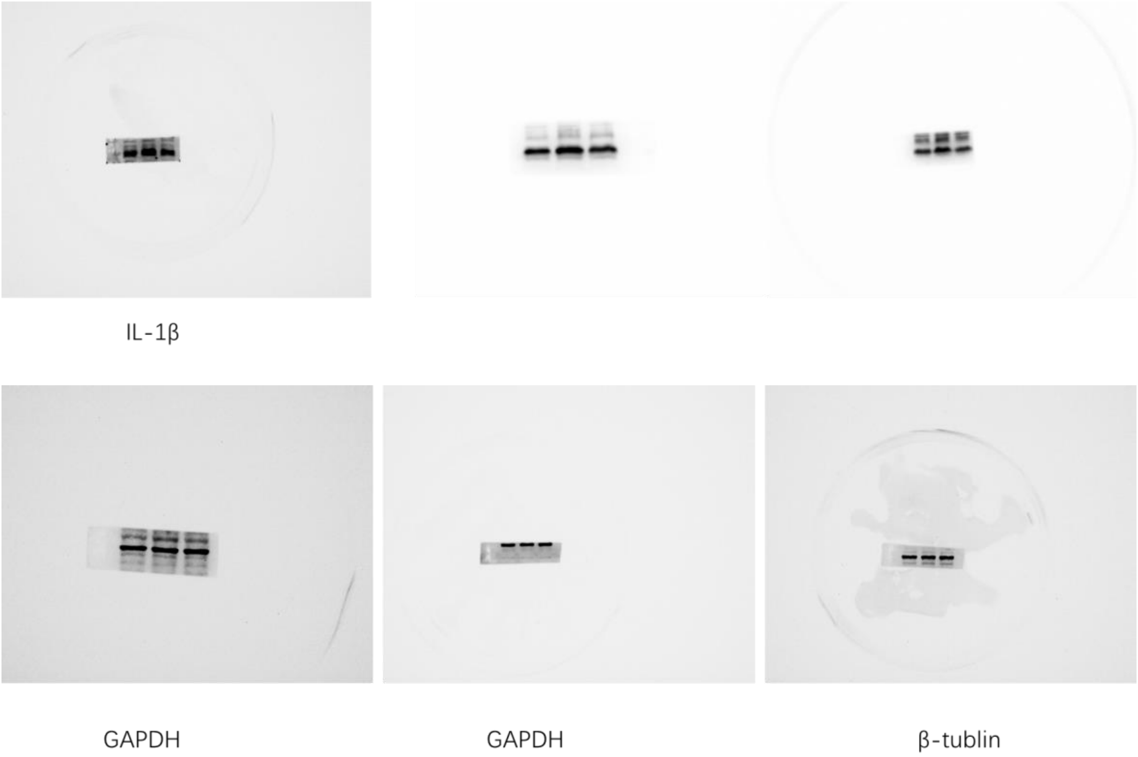

Figures 7. Uncropped western blot of the original image of Figures 5J in the paper.
